# Supplementary material for: Complexome profiling of the Chlamydomonas psb28 mutant reveals TEF5 as an early PSII assembly factor
Source: Plant Cell. 2025 Mar 17;37(6):koaf055. doi: 10.1093/plcell/koaf055 (PMC12164590; doi:10.1093/plcell/koaf055)
Supplement: koaf055_Supplementary_Data [file koaf055_supplementary_data.zip › psb28_interactive.zip!/plots/intensity/qualityPlots/peptides/MCP28_Cre15.g641200.t1.2.html]

### Cre15.g641200.t1.2

Trivial name: MCP28  
SeqCoverage [median]: 48.30  
Mass: 32.49 kDa  
  
 

| Sequence | MissedCleavage | Mass [Da] | wtIntensity | muIntensity | Score |
| --- | --- | --- | --- | --- | --- |
| AIELPTAATSEK | 0 | 1229.65 | 63444.85 | 114310.90 | 138.66 |
| ASGAGGAAAVFFIPK | 0 | 1362.73 | 45066.04 | 5185.17 | 233.54 |
| AVVAADGVAGLWK | 0 | 1255.69 | 3469.68 | 0.00 | 137.95 |
| GAMPGLIR | 0 | 813.45 | 0.00 | 788.34 | 94.16 |
| GFFFGGAR | 0 | 857.42 | 7017.67 | 1825.80 | 101.33 |
| KYAGLQSL | 1 | 878.49 | 3846.08 | 2924.51 | 97.07 |
| LGLYTPIK | 0 | 903.54 | 9366.43 | 28829.28 | 134.66 |
| LGPHTVIMFLTAER | 0 | 1583.85 | 3821.82 | 7335.11 | 63.16 |
| LQAAGRDPTVPK | 1 | 1251.69 | 64827.20 | 119812.17 | 96.07 |
| NQLAAGVKPPGMIATGINVVR | 1 | 2105.18 | 34262.08 | 125140.06 | 113.47 |
| SDGLIGFMK | 0 | 966.48 | 14096.22 | 9091.55 | 107.90 |
| TEGVGALWSGLGPSLAR | 0 | 1669.88 | 2311.90 | 0.00 | 129.54 |
| TSVGVIR | 0 | 730.43 | 10742.45 | 56908.36 | 137.83 |
|  |  |  | 262272.4 | 472151.2 |  |
